# Inhibiting kinesin family member 20A disrupts Zika virus entry by blocking internalization

**DOI:** 10.71150/jm.2503008

**Published:** 2025-06-30

**Authors:** Jeonghyeon Lee, Younghyun Lim, Hyeong-Rae Kim, Yong-Bin Cho, In-Gu Lee, Young-Jin Seo

**Affiliations:** Department of Life Science, Chung-Ang University, Seoul 06974, Republic of Korea

**Keywords:** Zika virus, kinesin, KIF20A, antiviral therapy

## Abstract

Zika virus, a mosquito-borne virus, is associated with congenital birth defects and neurological complications. However, despite its significant public health threat, no approved vaccines or antiviral treatments are currently available. Therefore, this study aims to identify kinesin family member 20A as a key host factor promoting Zika virus life cycle. The elevated expression of kinesin family member 20A following Zika virus infection suggests its role in the viral life cycle. Suppressing its expression through gene silencing or inhibiting its function with a small-molecule inhibitor significantly reduced viral infectivity in host cells. Furthermore, kinesin family member 20A is essential for facilitating viral internalization, a key step in the entry step. These findings suggest its significance in the Zika virus life cycle and highlight its potential as a novel therapeutic target for the Zika virus.

## Introduction

Zika virus (ZIKV) is a mosquito-borne flavivirus first identified in Uganda in 1947, which has since gained significant global attention owing to its rapid transmission and severe clinical manifestations (Weaver et al., 2016). ZIKV infection has been strongly associated with congenital birth defects, particularly microcephaly (Brasil et al., 2016), and neurological disorders such as Guillain–Barré syndrome (Fauci and Morens, 2016). Extensive research shows key aspects of ZIKV biology, including its pathogenesis and cellular tropism, particularly its ability to infect neural progenitor cells and placental tissues (Miner and Diamond, 2017; Tajik et al., 2024). However, despite significant advances in understanding the ZIKV life cycle, no effective antiviral therapy is currently available. Traditional approaches that directly target the virus face challenges owing to its genetic variability, including multiple serotypes and rapid mutation rates, which complicate the development and clinical validation of the therapeutics. Targeting host proviral factors presents a promising alternative, as it could offer a more effective therapeutic strategy to mitigate the effect of viral genetic variability, including that of ZIKV (Bekerman and Einav, 2015).

Cytoskeletal transport systems are essential for the intracellular trafficking of proteins, lipids, and organelles (Hirokawa et al., 2009). Many viruses exploit these systems to facilitate key stages of their life cycles, particularly by hijacking microtubule-based motor proteins such as kinesins (Malikov et al., 2015). These ATP-dependent molecular motors transport cargo along microtubules and are essential for intracellular vesicle trafficking, cell division, and organelle positioning (Gennerich and Vale, 2009). Kinesins contribute to multiple stages of viral life cycle, including entry, assembly, and egress. For example, kinesin family members 11, 13, 18A, and 20A (KIF20A) function as proviral host factors for the influenza virus (Cho et al., 2020; Jeon et al., 2022; Kim et al., 2021; Ramos-Nascimento et al., 2017). Similarly, kinesin family member 5 is involved in the life cycle of herpes simplex virus and human immunodeficiency virus (HIV) (DuRaine et al., 2018; Lukic et al., 2014). Kinesin family member 3 is implicated in the replication of HIV and Kaposi’s sarcoma-associated herpesvirus (Sathish et al., 2009). Given their essential roles in viral propagation, kinesins represent promising therapeutic targets for the development of novel antiviral therapies.

KIF20A is a motor protein that harnesses energy from ATP hydrolysis to facilitate microtubule-dependent transport of intracellular vesicles and cargo (She et al., 2020). KIF20A plays significant roles in cellular processes, particularly during mitosis and cytokinesis (Wu et al., 2019; Zhang et al., 2014). KIF20A and its regulatory proteins—such as Polo-like kinase 1 (Plk1), Aurora Kinase B, and RhoA-associated coiled-coil-containing protein kinase 1—are known to contribute to the effective replication of various viruses, including influenza, hepatitis B (HBV), hepatitis C (HCV), and dengue viruses (DENV) (Diab et al., 2017; Jeon et al., 2022; Madejon et al., 2015; Pérez-Olais et al., 2019). This suggests that KIF20A plays a crucial role in facilitating viral life cycle of a wide range of viruses by modulating key cellular processes.

Given its role in multiple viral infections, KIF20A may also regulate cellular processes during ZIKV infection. However, its specific contribution to ZIKV infection remains unclear. Therefore, this study aims to investigate the potential role of KIF20A in ZIKV life cycle.

## Materials and Methods

### Cell lines

Vero E6 (African green monkey kidney), HEK293T (human embryonic kidney), and Huh-7 (human hepatoma) cells were cultured in Dulbecco’s Modified Eagle Medium (DMEM; GenDEPOT, USA), supplemented with 10% fetal bovine serum (FBS; GenDEPOT, USA) and 1% penicillin-streptomycin (Welgene, Korea), at 37°C in a humidified incubator with 5% CO₂.

### Virus propagation and infection

The ZIKV strain (ZIKV/Homo sapiens/PRI/PRVABC59/2015; VR-1843, ATCC, USA) was used for all experiments. The virus was propagated in Vero E6 cells, and viral titers were quantified using a focus-forming assay. For infections, Vero E6, HEK293T or Huh-7 cells were incubated with ZIKV for 1 h in DMEM supplemented with 2% heat-inactivated fetal bovine serum GenDEPOT and 1% penicillin-streptomycin (Welgene), with gentle rocking every 10 min.

### Reagents

Paprotrain (≥ 97%), a KIF20A inhibitor, was purchased from Tocris Bioscience (UK) and dissolved in dimethyl sulfoxide (DMSO; Sigma-Aldrich, USA) at a concentration of 20 mM. Chloroquine was purchased from Sigma-Aldrich and dissolved in phosphate-buffered saline (PBS) at a concentration of 50 mM. Ribavirin was purchased from KisanBio (Korea) and dissolved in phosphate-buffered saline (PBS) at a concentration of 50 mM. Dynasore was purchased from Sigma-Aldrich and dissolved in phosphate-buffered saline (PBS) at a concentration of 50 mM. Proteinase K was purchased from Biofiat (Korea) and dissolved in phosphate-buffered saline (PBS) at a concentration of 20 mg/ml.

### RNA silencing

Vero E6 cells were seeded at 2 × 10⁵ cells/well in 6-well plates and incubated overnight. For targeted gene knockdown, synthesized small interfering RNAs (siRNAs; Bionics, Korea) targeting KIF20A mRNA or a scrambled control siRNA were transfected into the cells using Lipofectamine^TM^ 3000 (Invitrogen, USA) following the instructions of the manufacturer. siRNA Sequences were siKIF20A #1 - GUUCUCAGCCAUUGCUAGCTT, siKIF20A #2 - CCCUUAUGCCCGGAUCCUATT, siKIF20A #3 - GUCGUAGUUUCUCCCAUGUTT. At 72 h post-transfection, the transfection medium was replaced with a fresh culture medium while the cells were incubated overnight. The following day, the cells were infected with ZIKV at a multiplicity of infection (MOI) of 5. At the indicated time points, KIF20A mRNA and viral RNA expression levels were analyzed using real-time quantitative reverse transcription PCR (qRT-PCR).

### Generation of stable cell lines

A stable HEK293T cell line with KIF20A mRNA knockdown was generated using lentiviral transduction. For lentiviral production, HEK293T cells were seeded in a lentivirus packaging medium (Opti-MEM, 5% FBS, and 1x sodium pyruvate) at a density of 1 × 10^6^ cells/well in 6-well plates. After 24 h, the cells were co-transfected with lentiviral packaging vectors (shRNA KIF20A, shRNA scramble, psPAX2, and pMD2.G) using Lipofectamine^TM^ 3000 (Invitrogen). After 3 h, the culture medium was replaced with lentivirus boosting medium (Opti-MEM, 5% FBS, 1× sodium pyruvate, and 10 mM sodium butyrate). After an additional 6 h, the lentivirus boosting medium was replaced with a lentivirus packaging medium. The viral supernatant was collected twice, at 24 h and 48 h post-transfection. The lentiviral harvest medium was filtered using Corning^®^ syringe filters (0.45 µm; USA) and stored at 4°C. For lentiviral transduction, HEK293T cells were incubated with lentivirus infection medium in the presence of 8 µg/ml polybrene. At 48 h post-transduction, the cells were selected with 1 µg/ml puromycin (Sigma-Aldrich) for 3 days.

### Real-time quantitative reverse transcription polymerase chain reaction

Total RNA was isolated from cells using NucleoZOL (Macherey-Nagel, Germany) following the instructions of the manufacturer. The isolated RNA was reverse-transcribed using TOPscript^TM^ RT DryMIX (Enzynomics, Korea). qRT-PCR was performed using the CFX Connect^TM^ Real-Time PCR Detection System (Bio-Rad Laboratories, USA) under the following cycling conditions: 95°C for 15 min, followed by cycles of 55°C for 20 s and 73°C for 20 s, with a final melting curve analysis. The following primers were used in the mRNA quantification protocol: for NS3, forward 5’-TGC CAT GCC ACC TTC ACT TCA C-3’ and reverse 5’-CCT CGC CCA TCT CAA CCC TTG T-3’; for E, forward 5’-GGG TTG ATG TTG TCT TGG AAC AT-3’ and reverse 5’-AGG CTT CAC CTT GTG TTG GG-3’; for KIF20A, forward 5’-ACC AGC AGA ACC GGT CAA AG-3’ and reverse 5’-GCC TCG GCC TGT GAA GAA AC-3’; and for GAPDH, forward 5’-TGG ACC TGA CCT GCC GTC TA-3’ and reverse 5’-CCC TGT TGC TGT AGC CAA ATT C-3’. The following primers were used in the vRNA quantification protocol: for NS3, forward 5’-TGC CAT GCC ACC TTC ACT TCA-3’ and reverse 5’-CCT CGC CCA TCT CAA CCC TTG-3’; and for E, forward 5’-GGG TTG ATG TTG TCT TGG AAC AT-3’ and reverse 5’-AGG CTT CAC CTT GTG TTG GG-3’.

### Western blot analysis

Cells were lysed in NP-40 protein lysis buffer (Elpisbio, Korea) supplemented with a protease/phosphatase inhibitor cocktail (Thermo Fisher Scientific). Subsequently, the lysates were separated by 10% sodium dodecyl sulfate-polyacrylamide gel electrophoresis (SDS-PAGE) and transferred onto nitrocellulose membranes. The membrane was first blocked with 3% bovine serum albumin (BSA) in Tris-buffered saline with Tween-20 (TBST) for 1 h at room temperature, followed by overnight incubation at 4°C with primary antibodies diluted in 3% BSA/TBST. The primary antibodies used were anti-Actin (1:200; Cell Signaling Technology, USA), anti-β Tubulin (1:200; Santa Cruz Biotechnology, USA) and anti-ZIKV Envelope protein (1:1000; GeneTex, USA). After washing, the membrane was incubated with horseradish peroxidase (HRP)-conjugated secondary antibodies in 3% BSA/TBST for 1 h at room temperature. Protein bands were detected using the SuperSignal^TM^ West Pico PLUS Enhanced Chemiluminescent Substrate (Thermo Fisher Scientific) and quantified with the FluorChem E Imaging System (ProteinSimple, USA).

### Immunofluorescence

Vero E6 and Huh-7 cells were seeded at 2 × 10⁵ cells/well in 4-well chamber slides (Millipore, USA) and incubated overnight. Subsequently, the cells were infected with ZIKV at an MOI of 5 for 1 h. At the indicated time points, the cells were fixed with 4% paraformaldehyde (PFA) in PBS for 20 min at room temperature, permeabilized with 0.5% Triton^TM^ X-100 in PBS for 10 min, and blocked with 1% FBS in PBS for 2 h at room temperature. Primary antibodies (anti-ZIKV Envelope protein antibody, 1:1000 dilution) were applied overnight at 4°C. Following three washes with PBS, the cells were incubated with Alexa Fluor^TM^ 488-conjugated goat anti-rabbit immunoglobulin G (IgG) (1:500; Thermo Fisher Scientific) for 1 h at room temperature. The nuclei were stained using 4′,6-diamidino-2-phenylindole (DAPI) mounting solution (Fluoroshield^TM^ with DAPI; Sigma-Aldrich). Images were captured using an LSM710 confocal microscope (Carl Zeiss, Germany).

### Intracellular flow cytometry analysis

Vero E6 and Huh-7 cells were seeded at a density of 2 × 10⁵ cells/well in 24-well plates and incubated overnight. Subsequently, the cells were infected with ZIKV at an MOI of 5 for 1 h. Following infection, the cells were fixed and permeabilized using the Cyto-Fast^TM^ Fix-Perm Buffer Set (BioLegend, USA). Intracellular viral proteins were detected by staining with an anti-ZIKV Envelope protein antibody (1:5000; GeneTex), followed by incubation with an Alexa Fluor^®^ 488-conjugated goat anti-rabbit IgG secondary antibody (1:50,000; Thermo Fisher Scientific). All samples were analyzed using FlowJo v.10 software (BD Biosciences, USA).

### Focus-forming assay

Vero E6 cells were seeded at a density of 1 × 10⁵ cells/well in 24-well plates, before being incubated after 24 h. The cells were exposed to supernatants from infected cultures at 37°C for 1 h. After incubation, the cells were overlaid with 0.8% methylcellulose (Sigma-Aldrich) in DMEM, supplemented with 2% heat-inactivated fetal bovine serum and 1% penicillin-streptomycin. After 3 days, the cells were fixed with 3.5% formaldehyde for 20 min at room temperature, permeabilized with 0.5% Triton X-100 in PBS for 10 min, and blocked with 1% FBS in PBS for 2 h at room temperature. Subsequently, the cells were incubated overnight at 4°C with a primary antibody against the anti-flavivirus group antigen (1:50000 dilution; Merck, USA) in 3% BSA in TBST. This was followed by incubation with the appropriate HRP-conjugated secondary antibodies for 1 h at room temperature. Viral foci were visualized using the Metal-Enhanced DAB Substrate Kit (Thermo Fisher Scientific), appearing as dark purple spots upon visual inspection.

## Results

### Zika virus infection increases the expression of KIF20A

**Fig. 1.**
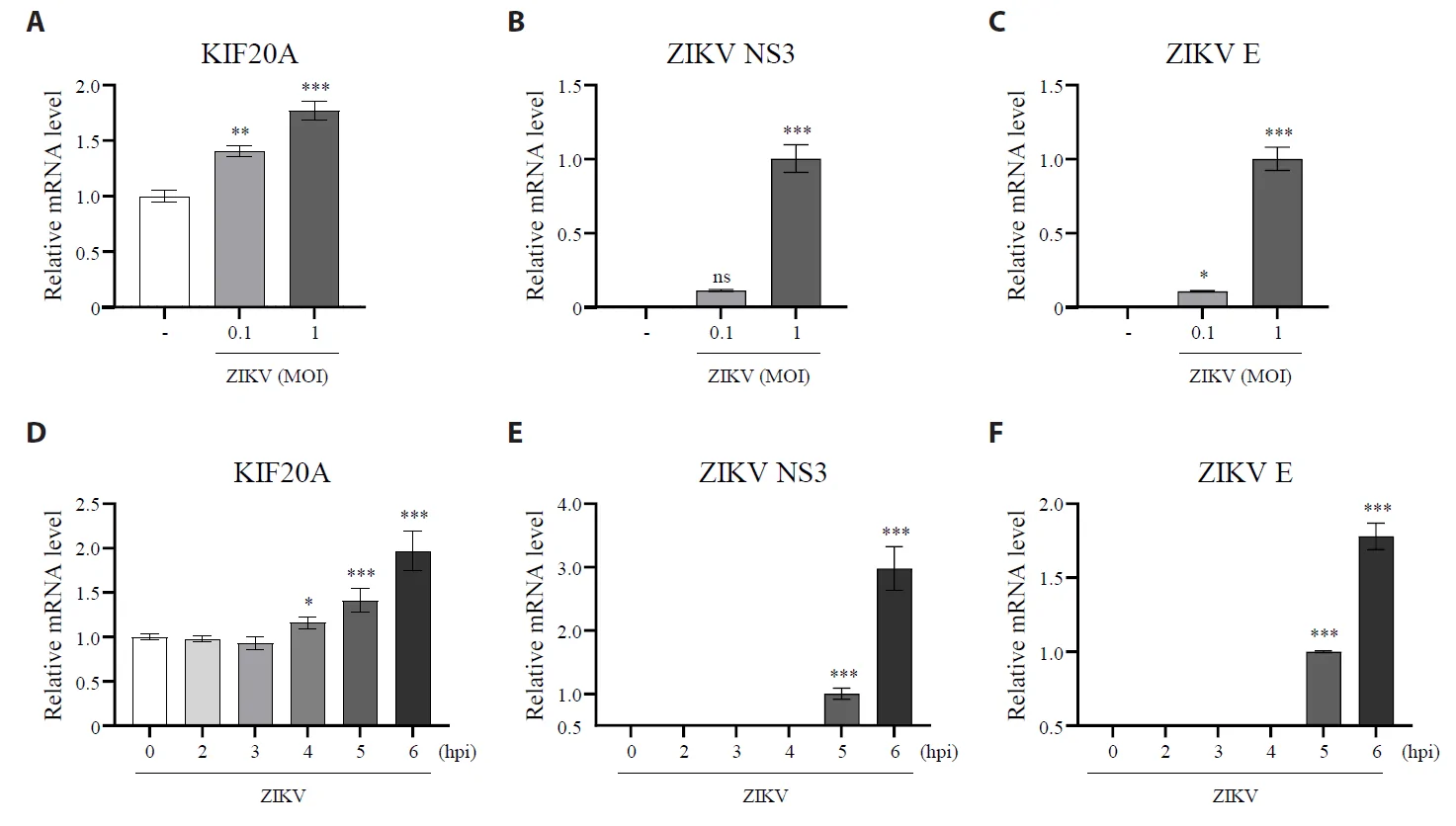
Zika virus infection increases the expression of KIF20A. (A–C) Vero E6 cells were infected with Zika virus (ZIKV) at a multiplicity of infection (MOI) of 0.1 or 1. Cells were harvested at 6 h post-infection (hpi), and mRNA levels of KIF20A, ZIKV NS3, and ZIKV E were quantified using qRT-PCR. (A) Relative KIF20A mRNA levels normalized to GAPDH. (B, C) Relative mRNA levels of ZIKV NS3 (B) and ZIKV E (C) normalized to GAPDH. (D–F) Vero E6 cells were infected with Zika virus (ZIKV) at a multiplicity of infection (MOI) of 1. Cells were harvested at 0, 2, 3, 4, 5, and 6 h post-infection, and mRNA levels of KIF20A, ZIKV NS3, and ZIKV E were quantified using qRT-PCR. (D) Relative KIF20A mRNA levels normalized to GAPDH. (E, F) Relative mRNA levels of ZIKV NS3 (E) and ZIKV E (F) normalized to GAPDH. Data are expressed as the mean ± standard error of the mean (SEM). All experiments were performed at least twice. Asterisks indicate statistically significant differences as determined using an analysis of variance (ANOVA) (^**^P < 0.01, ^***^P < 0.001).

A previous study reported that KIF20A is a proviral factor for influenza virus, an RNA virus (Jeon et al., 2022). Based on this finding, we investigated whether KIF20A played a similar role in the infection of other RNA viruses, specifically ZIKV. To assess the relationship between viral input and host response, we used Vero E6 cells, which are highly permissive to ZIKV and lack type I interferon responses, were used to ensure consistent viral amplification and reliable measurement of host gene expression (Ramos da Silva et al., 2019). Vero E6 cells were infected with ZIKV at MOIs of 0.1 or 1, and KIF20A mRNA levels were measured (Fig. 1A). KIF20A expression levels were positively correlated with both the MOIs of ZIKV and intracellular ZIKV RNA levels (Fig. 1A–1C). Moreover, KIF20A mRNA levels exhibited a significant increase beginning at 5 h post-infection (hpi) (Fig. 1D; 21% increase at 5 hpi relative to 4 hpi, and an additional 40% increase at 6 hpi relative to 5 hpi). Similarly, the mRNA levels of the ZIKV Envelope protein (E) and Non-Structural protein 3 (NS3) increased at 6 hpi (Fig. 1E; 291% increase at 6 hpi compared to 5 hpi, Fig. 1F; 77% increase at 6 hpi compared to 5 hpi). These findings suggest that KIF20A may contribute to ZIKV replication.

### KIF20A knockdown attenuates Zika virus infection

**Fig. 2.**
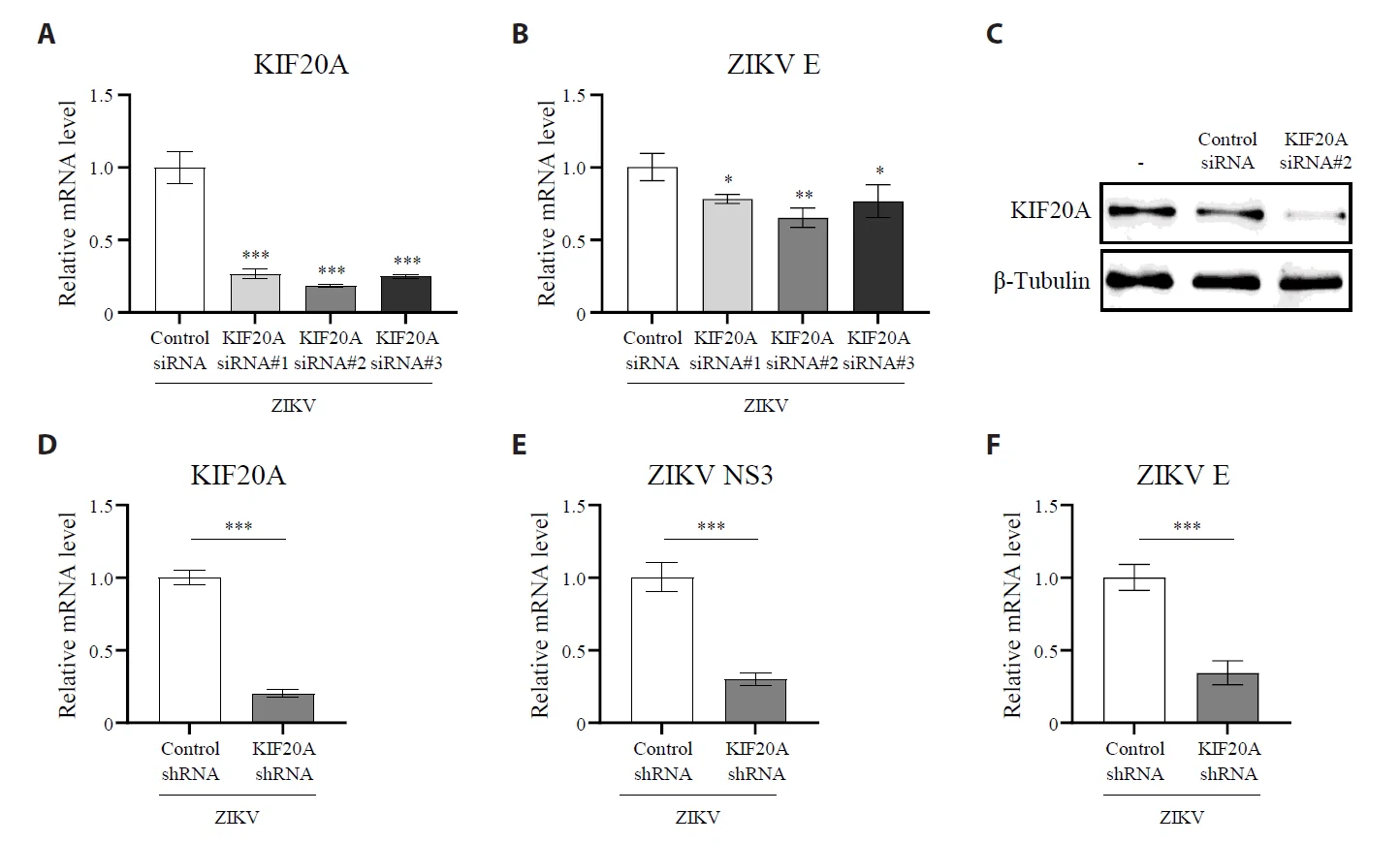
KIF20A knockdown attenuates Zika virus infection. (A–C) Vero E6 cells were transfected with either control scrambled small interfering RNA (siRNA) or one of three different siRNA targeting KIF20A (siRNA #1, #2, and #3). After transfection, cells were infected with Zika virus (ZIKV) at a multiplicity of infection (MOI) of 5. At 6 h post-infection (hpi), total RNA was extracted, while the mRNA levels of KIF20A and ZIKV NS3 were quantified using qRT-PCR. (A) Relative KIF20A expressions normalized to GAPDH. (B) Relative mRNA levels of ZIKV E normalized to GAPDH. (C) KIF20A protein levels analyzed using western blot. (D–F) HEK293T cells were transduced with lentivirus encoding either control shRNA or KIF20A-targeting shRNA. Transduction efficiency was confirmed using a GFP reporter and selected with puromycin. Stable cell lines were infected with ZIKV at an MOI of 0.5. At 6 hpi, total RNA was extracted, while the mRNA levels of KIF20A, ZIKV NS3, and ZIKV E were quantified. (D) Relative mRNA levels of KIF20A normalized to GAPDH. (E, F) Relative mRNA levels of ZIKV NS3 (E) and ZIKV E (F) normalized to GAPDH. Values are expressed as the mean ± standard error of the mean (SEM). All experiments were performed at least twice. Asterisks indicate statistically significant differences using an as determined using an analysis of variance (ANOVA) (^*^P < 0.1, ^**^P < 0.01, ^***^P < 0.001), or unpaired t-test (^***^P < 0.001).

Given that ZIKV infection increased KIF20A expression (Fig. 1), we next analyzed whether KIF20A downregulation affected ZIKV life cycle. To this end, Vero E6 cells were transfected with three different KIF20A-targeting siRNA or scrambled control siRNA. KIF20A siRNA efficiently silenced mRNA levels by > 70%, compared to that of the control (Fig. 2A), leading to a significant reduction in the mRNA expression of ZIKV E (Fig. 2B; Control siRNA:1.0 vs. KIF20A siRNA #1: 0.78, KIF20A siRNA #2 0.65, KIF20A siRNA #3 0.76). Among these, siRNA #2 exhibited the most robust and reproducible knockdown effect. We therefore selected this siRNA for further analysis and confirmed its efficacy at the protein level by Western blotting, which clearly showed the reduced KIF20A protein expression (Fig. 2C). These results were further validated using a lentiviral shRNA knockdown system. HEK293T cells with stable KIF20A knockdown via lentiviral transduction (Fig. 2D) also exhibited a significant reduction in ZIKV mRNA expression (Fig. 2E; Control shRNA: 1.0 vs. KIF20A shRNA: 0.3, Fig 2F; Control shRNA: 1.0 vs. KIF20A shRNA: 0.34). Taken together, these findings indicate that KIF20A is a proviral factor for ZIKV.

### KIF20A inhibition suppresses Zika virus infection

**Fig. 3.**
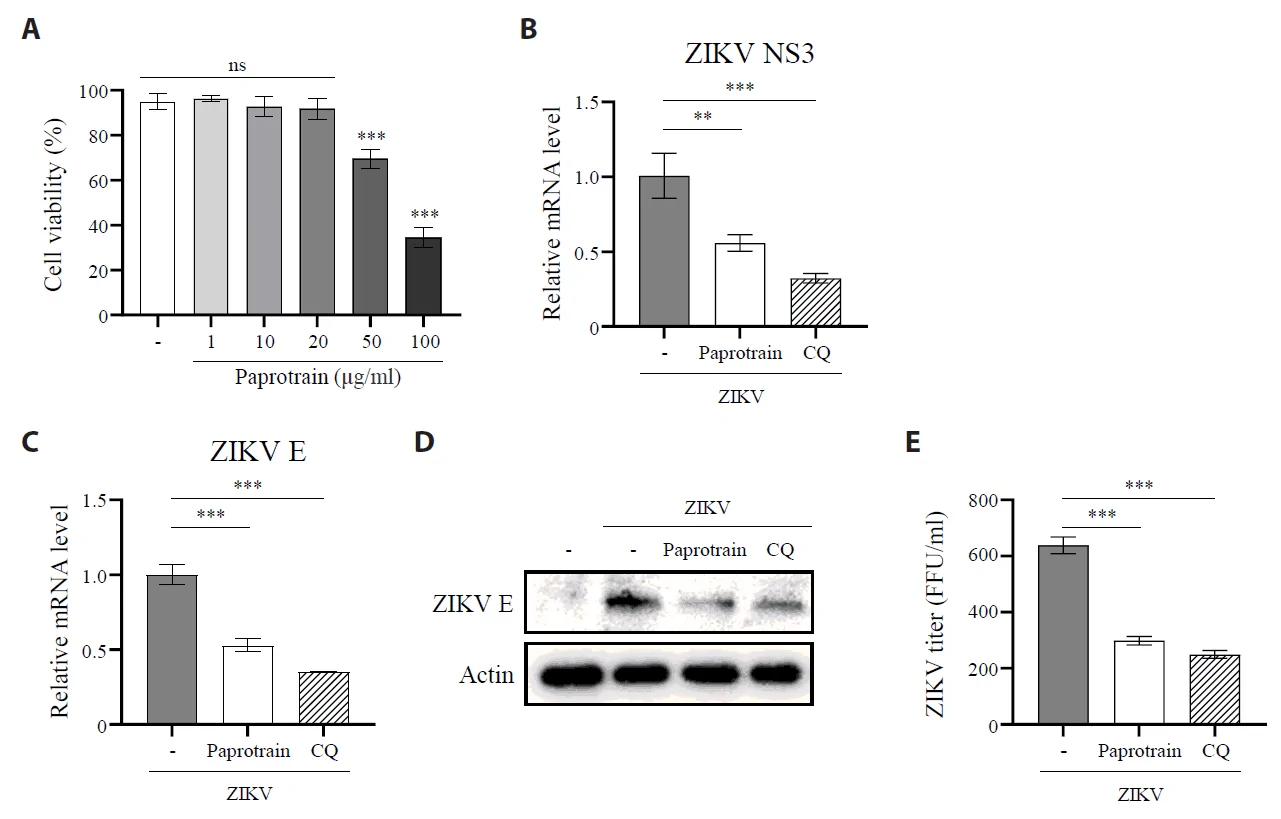
KIF20A inhibition suppresses Zika virus infection. (A) Vero E6 cells were treated with paprotrain at concentrations ranging from 100 to 1 µg/ml. After 12 h, cell viability was determined using the trypan blue exclusion assay. (B–E) Vero E6 cells were infected with ZIKV at a multiplicity of infection (MOI) of 0.5 and co-treated with either 20 µg/ml paprotrain or 50 µM chloroquine (CQ) for 18 h. (B, C) Relative mRNA levels of ZIKV NS3 (B) and E (C) normalized to GAPDH. (D) ZIKV E protein levels analyzed using Western blot. (E) Viral titers in the culture medium quantified using a focus-forming assay. Values are expressed as the mean ± standard error of the mean (SEM). All experiments were performed at least twice. Asterisks indicate statistically significant differences as determined using an analysis of variance (ANOVA) (^**^P < 0.01, ^***^P < 0.001).

Since KIF20A knockdown interfered with ZIKV infection (Fig. 2), we hypothesized that inhibiting KIF20A activity might also suppress viral infection on host cells. To test this, paprotrain—a cell-permeable small-molecule inhibitor of KIF20A ATPase activity—was used (Tcherniuk et al., 2010). First, we determined the optimal paprotrain concentration that did not induce cytotoxicity in cell lines used in this study, ensuring that any decrease in ZIKV infectivity was not attributed to its cytotoxicity. Consistent with previous reports (Jeon et al., 2022; Zhu et al., 2020), high concentrations of paprotrain (50 or 100 μg/ml) induced cytotoxicity in Vero E6 (Fig. 3A; Viable cells: 97.5% at 0 μg/ml, 97.5% at 1 μg/ml, 93.8% at 10 μg/ml, 91.8% at 20 μg/ml, 68% at 50 μg/ml, and 35% at 100 μg/ml) cells. Therefore, all subsequent experiments were performed using paprotrain at concentrations of ≤ 20 μg/ml.

To examine whether the KIF20A inhibitor suppresses ZIKV infection on host cells, Vero E6 cells were infected with ZIKV and treated with paprotrain, followed by measuring viral RNA levels, protein expression, and viral titers. Before evaluating the antiviral effects of paprotrain, we first selected an appropriate positive control compound to establish a reliable dynamic range between negative and positive responses in our system. To this end, we tested ribavirin and chloroquine (CQ) that have been previously reported as RNA virus inhibitors (Kamiyama et al., 2017; Kim et al., 2018; Shiryaev et al., 2017; Yang et al., 2018). CQ exhibited more potent inhibitory effects against ZIKV than ribavirin (Fig. S1A–S1C). Therefore, we used CQ as a control compound in subsequent experiments. Consistent with the results in Fig. 2, paprotrain treatment significantly reduced the RNA expressions of ZIKV NS3 (Fig. 3B; untreated: 1.0 vs. paprotrain-treated: 0.55 vs. CQ-treated: 0.32) and ZIKV E (Fig. 3C; untreated: 1.0 vs. paprotrain-treated: 0.52 vs. CQ-treated: 0.35). Additionally, paprotrain treatment led to a significant reduction in viral protein expression (Fig. 3D) and the production of infectious viral particles (Fig. 3E; untreated: 638 vs. paprotrain-treated: 298 vs. CQ-treated: 249). Taken together, these findings indicate that inhibiting KIF20A activity by paprotrain effectively suppresses ZIKV infection on host cells.

### KIF20A inhibitor treatment reduced the number of Zika virus-infected cells

**Fig. 4.**
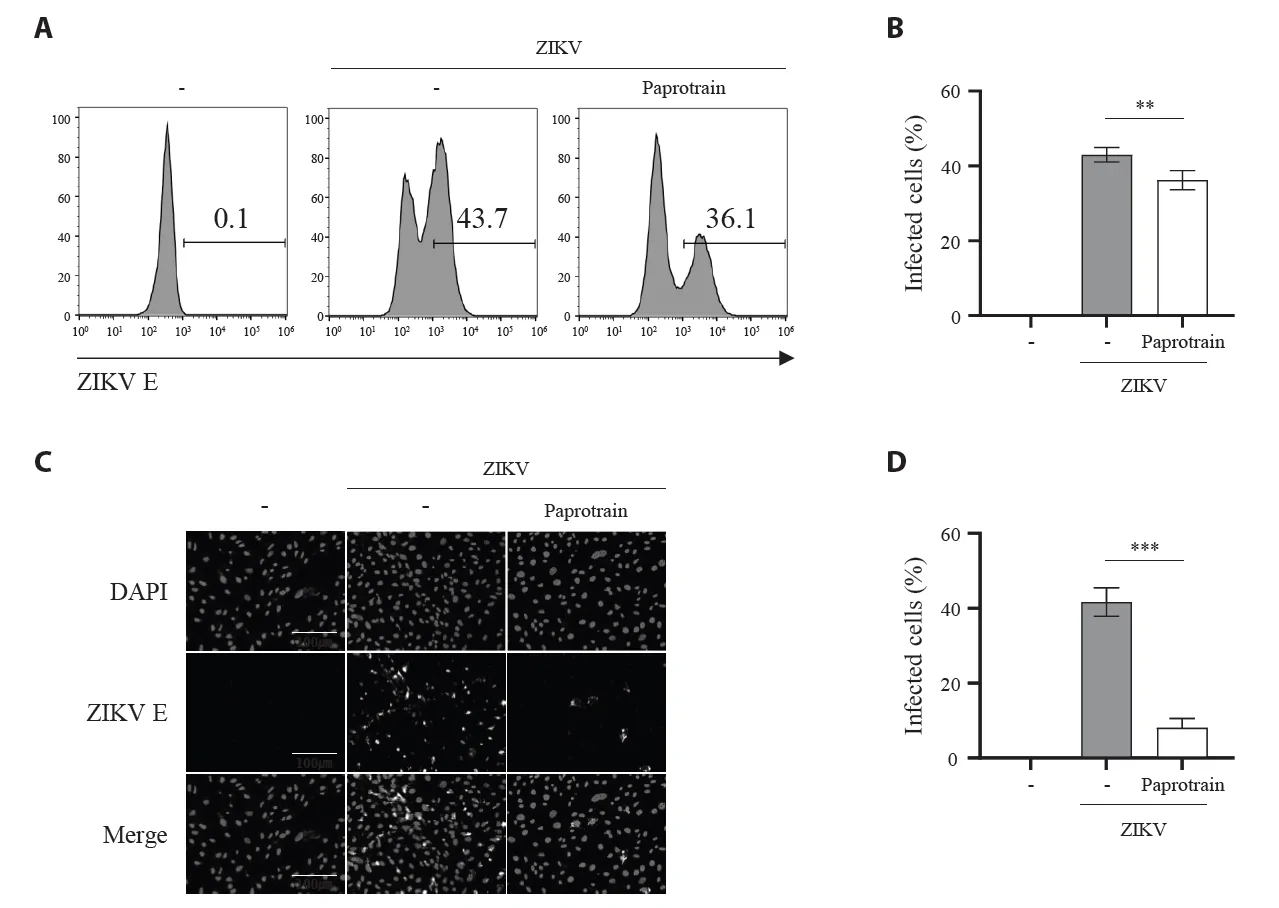
KIF20A inhibition reduces the number of ZIKV-infected cells. (A, B) Vero E6 cells infected with ZIKV at a multiplicity of infection (MOI) of 5, with or without 20 µg/ml paprotrain. After 18 hpi, infected cells were fixed, permeabilized, and stained with an anti-ZIKV E protein antibody for flow cytometric analysis. (A) Representative histograms illustrating the proportion of ZIKV E–positive (infected) cells. (B) Percentages of infected cells. (C, D) Vero E6 cells infected with ZIKV at an MOI of 5, with or without 20 µg/ml paprotrain. After 18 hpi, infected cells were fixed, permeabilized, and stained with DAPI and an anti-ZIKV E protein antibody for immunofluorescence microscopy. (C) Representative images showing infected cells. (D) Quantification from three independent experiments, where infected cells were defined as the number of ZIKV E–positive nuclei (co-localized with DAPI) divided by the total number of DAPI–stained nuclei. Values are expressed as the mean ± standard error of the mean (SEM). All experiments were performed at least twice. Asterisks indicate statistically significant differences as determined using an analysis of variance (ANOVA) (^*^P < 0.01, ^***^P < 0.001).

Since our results demonstrated that KIF20A inhibition decreased the overall viral RNA expression, viral protein levels, and virus titers, we further examined whether inhibiting KIF20A could also reduce the number of ZIKV-infected cells. To address this, we performed intracellular flow cytometry analysis to evaluate infection at the single-cell level. Vero E6 cells infected with ZIKV were left untreated or treated with paprotrain, followed by flow cytometry analysis. Paprotrain-treated Vero E6 cells exhibited a significantly lower percentage of infected cells (Fig. 4A and 4B; untreated: 43.7 vs. paprotrain-treated: 36.1). Consistent with these results, immunofluorescence imaging analysis revealed that paprotrain treatment significantly reduced the number of infected cells in Vero E6 cells (Fig. 4C and 4D; untreated: 41.7 vs. Paprotrain-treated: 8). Additionally, we observed a similar reduction in the percentage of infected cells upon paprotrain treatment in Huh-7 cells, confirming that the antiviral effect associated with KIF20A inhibition extends to other cell types (Fig. S2). These findings indicate that KIF20A inhibition reduces the proportion of cells susceptible to ZIKV infection, supporting its potential as a therapeutic host-targeting strategy.

### KIF20A inhibition attenuates Zika virus life cycle at an early time point

**Fig. 5.**
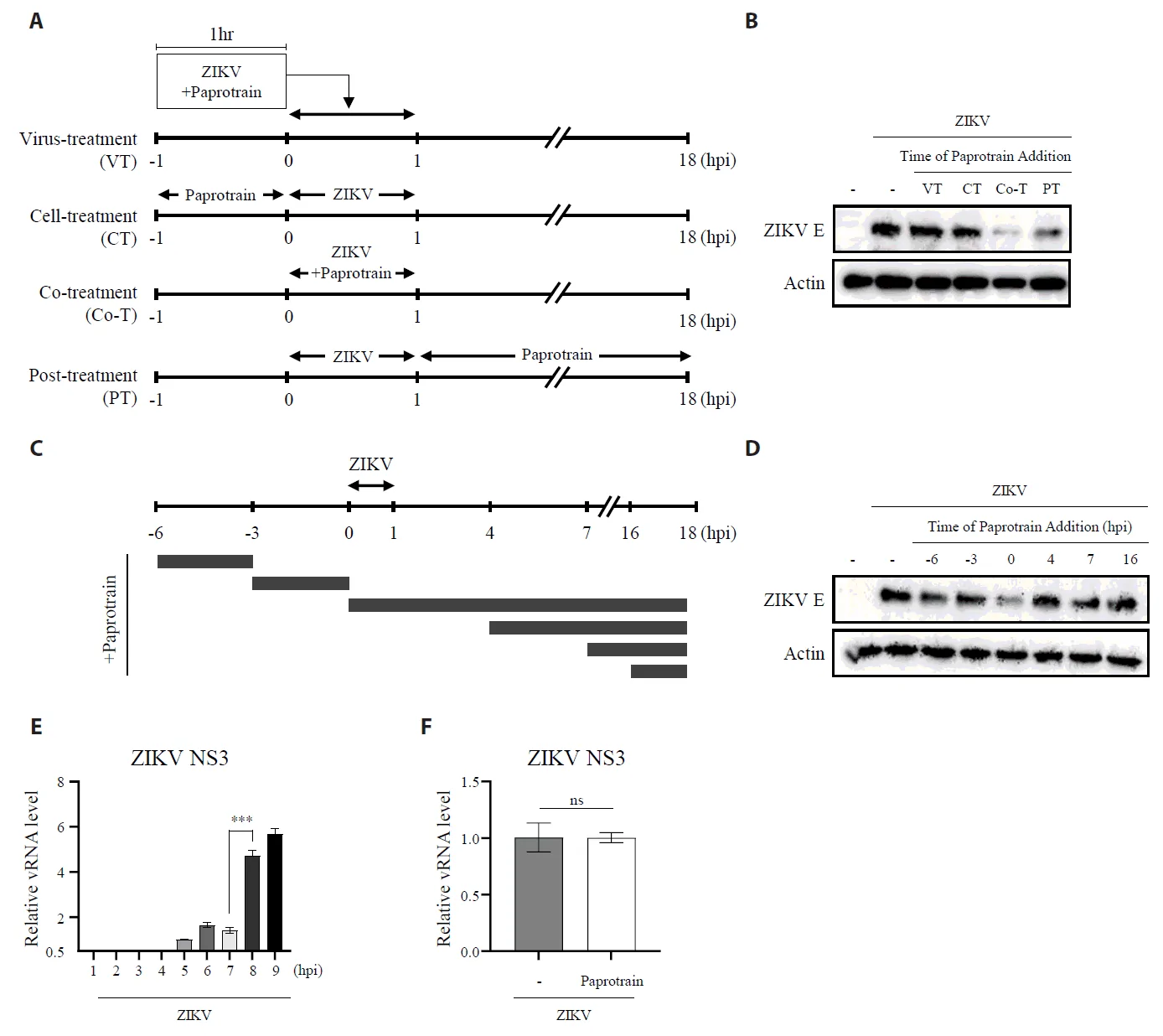
KIF20A inhibition attenuates Zika virus infection on host cells at an early time point. (A) Schematic representation of treatment conditions: virus treatment (VT), cell treatment (CT), co-treatment (Co-T), and post-treatment (PT). (B) Vero E6 cells infected with ZIKV at a multiplicity of infection (MOI) of 0.5. At 18 h post-infection (hpi), cells were harvested and lysed, and ZIKV E protein levels were analyzed using Western blot. (C) Schematic diagram of the time-of addition assay. Cells were treated with 20 µg/ml paprotrain at various time points relative to ZIKV infection. For pretreatment, cells received paprotrain 6 h and 3 h before infection. For co-treatment and post-treatment, paprotrain was added at 0 h (co-treatment) or 4 h, 7 h, and 16 h post-infection. (D) Vero E6 cells were infected with ZIKV at an MOI of 0.5. At 18 hpi, cells were harvested and lysed, while ZIKV E protein levels were analyzed using Western blot. All experiments were performed at least twice. (E) Vero E6 cells were infected with ZIKV at an MOI of 1, and relative vRNA levels of ZIKV NS3 were normalized to GAPDH at each time point after ZIKV infection. (F) Vero E6 cells were infected with ZIKV at an MOI of 1, and treated with 20 µg/ml paprotrain at 7 hpi for 1 h. Relative vRNA levels of ZIKV NS3 were normalized to GAPDH. Values are expressed as the mean ± standard error of the mean (SEM). All experiments were performed at least twice. Asterisks indicate statistically significant differences as determined using an analysis of variance (ANOVA) (^***^P < 0.001).

To identify the antiviral mechanisms of paprotrain, we conducted experiments under various treatment conditions (Fig. 5A). To determine whether paprotrain directly inhibited the ability of viral particles to infect host cells, ZIKV was preincubated with paprotrain for 1 h before infecting Vero E6 cells (virus treatment, VT), but no reduction in ZIKV E protein was observed (Fig. 5B). To assess whether paprotrain protects cells from viral infection, cells were pretreated with paprotrain for 1 h, washed, and subsequently infected with ZIKV for an additional hour (cell treatment, CT), but preincubation of cells with paprotrain did not reduce ZIKV E protein expression (Fig. 5B). In contrast, co-treatment (Co-T) and post-treatment with paprotrain significantly inhibited ZIKV E protein expression (Fig. 5B). These findings suggest that paprotrain suppresses ZIKV infection during viral entry or subsequent stages of the viral life cycle.

To further investigate the specific stages of the ZIKV life cycle affected by KIF20A inhibition, we performed a time-of-addition assay with varying paprotrain treatment timings (Fig. 5C). For the pretreatment condition, cells were pretreated with paprotrain for 6 (-6 hpi) or 3 (-3 hpi) h, followed by washed out and subsequent ZIKV infection. Consistent with the result in Fig. 5B, the reduction in viral protein expression was most pronounced when cells were co-treated (0 hpi) with paprotrain, while treatments at other time points showed comparatively lower effects (Fig. 5D). Additionally, we investigated whether the inhibition of KIF20A affects ZIKV RNA synthesis. As viral NS3 RNA levels increased markedly between 7 and 8 h post-infection (hpi) (Fig. 5E), Vero E6 cells were treated with paprotrain at 7 hpi for 1 h, followed by quantification of viral NS3 RNA (Fig. 5F). The results showed that paprotrain treatment did not significantly affect viral RNA synthesis. These findings suggest that KIF20A is not involved in ZIKV replication, but instead functions during the early stages of the ZIKV life cycle.

### Inhibition of KIF20A interferes with the Zika virus internalization during entry stages

**Fig. 6.**
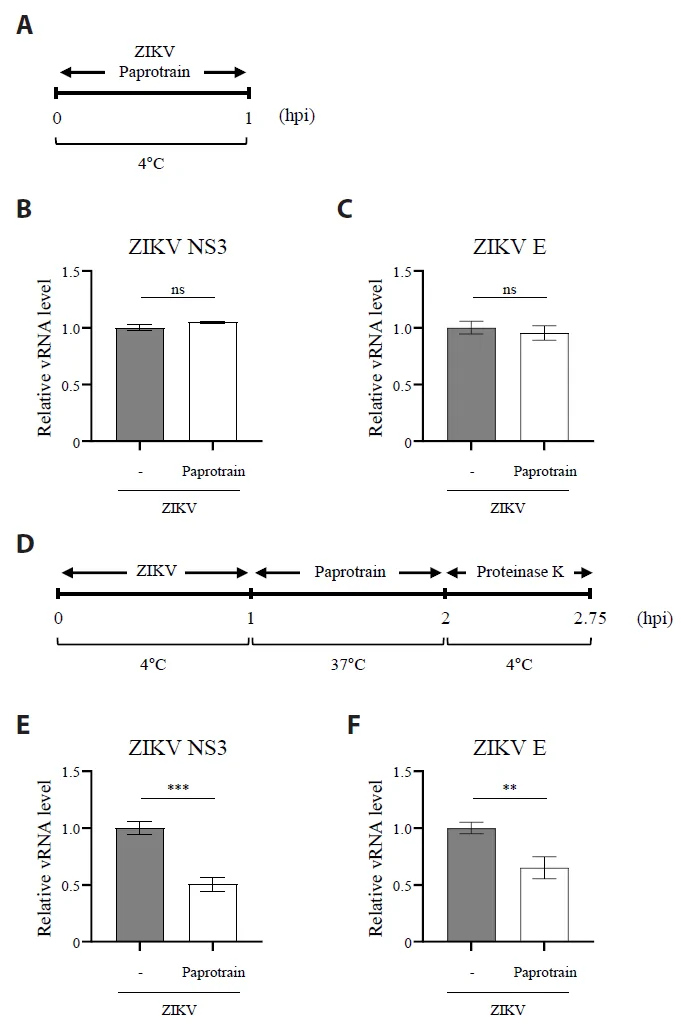
The inhibition of KIF20A interferes with the Zika virus internalization during entry stages. (A) Schematic representation of the experiment evaluating the effect of paprotrain on ZIKV attachment. (B, C) Vero E6 cells were infected with ZIKV at a multiplicity of infection (MOI) of 10 with or without 20 µg/ml paprotrain at 4°C. After 1 h, cells were washed five times with cold PBS. The samples were then immediately processed for total RNA extraction. Relative mRNA levels of ZIKV NS3 (B) and ZIKV E (C) normalized to GAPDH. (D) Schematic representation of the experiment evaluating the effect of paprotrain on ZIKV entry. (E, F) Vero E6 cells infected with ZIKV at a multiplicity of infection (MOI) of 10 for 1 h at 4°C. Following infection, cells were washed five times with cold PBS, followed by treatment with 20 µg/ml paprotrain. After incubation at 37°C for 1 h, the cells were washed with cold PBS and treated with proteinase K (1 mg/ml) for 45 min at 4°C to remove surface-bound but non-internalized virus. The samples were then immediately processed for total RNA extraction. Relative mRNA levels of ZIKV NS3 (E) and ZIKV E (F) normalized to GAPDH. Values are expressed as the mean ± standard error of the mean (SEM). All experiments were performed at least twice. Asterisks indicate statistically significant differences as determined using an analysis of variance (ANOVA) (^**^P < 0.01, ^***^P < 0.001).

We then aimed to identify the specific early-stage events in the viral life cycle affected by KIF20A inhibition. First, we examined whether paprotrain interfered with viral attachment to host cells. To assess this, Vero E6 cells were incubated with ZIKV at 4°C for 1 h, a condition that allowed viral attachment to the host cell surface (Fig. 6A). Cells were then thoroughly washed and lysed to quantify the attached virus. Interestingly, no significant differences in viral NS3 or E RNA levels were observed between untreated and paprotrain-treated conditions, indicating that ZIKV attachment was not affected by KIF20A inhibition (Fig. 6B and 6C).

Since the pronounced antiviral effect of paprotrain was observed when added into cells before 4 hpi (Fig 5D), it might affect the viral early entry stage following viral attachment. To test this, cells were incubated with ZIKV at 4°C for 1 h to allow virus attachment, followed by washing to remove unbound virus. The cells were then incubated at 37°C for 1 h to permit viral internalization, with or without paprotrain treatment. Subsequently, cells were treated with proteinase K to remove non-internalized virus (Fig. 6D). Treatment with the KIF20A inhibitor paprotrain during the early entry phase significantly reduced internalized virion (Fig. 6E; untreated: 1.0 vs. paprotrain-treated: 0.5, Fig. 6F; untreated: 1.0 vs. paprotrain-treated: 0.65). Taken together, these findings indicate that KIF20A may play a crucial role in facilitating ZIKV entry by internalization.

### Inhibition of KIF20A suppresses clathrin-mediated entry of ZIKV

**Fig. 7.**
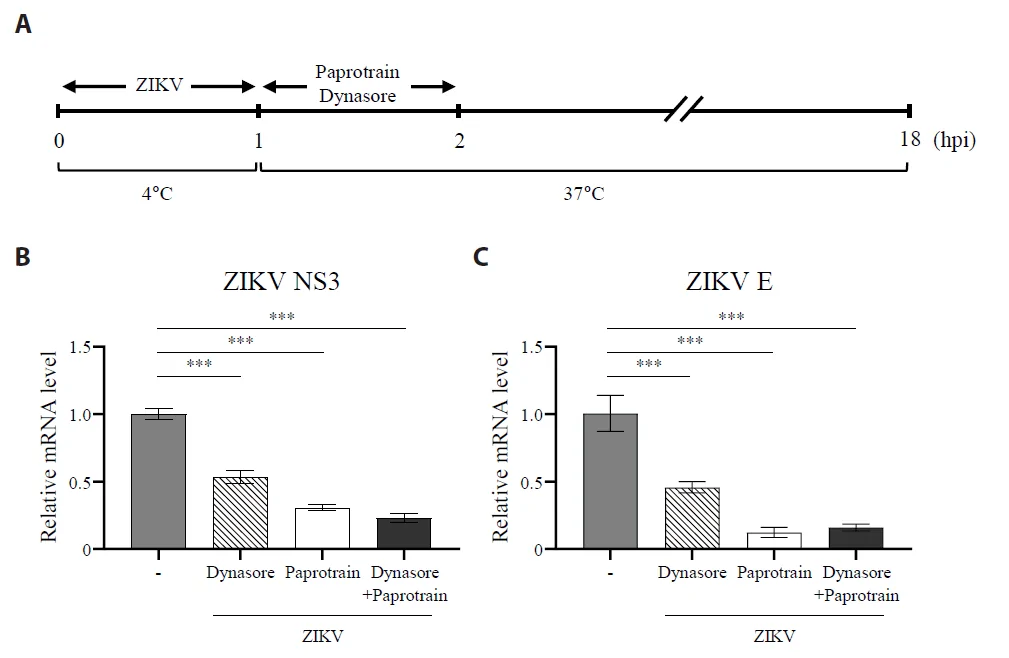
The inhibition of KIF20A suppresses clathrin-mediated entry of ZIKV. (A) Schematic representation of the experiment evaluating the effect of paprotrain on ZIKV clathrin-mediated endocytosis (CME) pathway. (B, C) Vero E6 cells were infected with ZIKV at a multiplicity of infection (MOI) of 0.5 at 4°C. After 1 h, cells were washed three times with PBS and subsequently treated with paprotrain (20 µg/ml), dynasore (50 µM), or a combination of paprotrain (20 µg/ml) and dynasore (50 µM). At 18 h post infection (hpi), the levels of viral RNA were measured. Relative mRNA levels of ZIKV NS3 (B) and ZIKV E (C) normalized to GAPDH. Values are expressed as the mean ± standard error of the mean (SEM). All experiments were performed at least twice. Asterisks indicate statistically significant differences as determined using an analysis of variance (ANOVA) (^**^P < 0.01, ^***^P < 0.001).

As ZIKV predominantly enters host cells via clathrin-mediated endocytosis (CME) (Persaud et al., 2018), we investigated whether KIF20A is associated with this pathway. Vero E6 cells were infected with ZIKV and treated with paprotrain, dynasore—a dynamin inhibitor that blocks vesicle scission in CME—or a combination of both during the internalization phase (Fig. 7A). As shown in Fig. 7B, paprotrain alone led to a greater reduction in ZIKV RNA levels than dynasore. Notably, the combined treatment did not result in a significantly enhanced inhibitory effect compared to paprotrain alone (Fig. 7B and 7C). These findings suggest that KIF20A and dynamin may function at overlapping stages of ZIKV internalization, potentially through a shared or converging mechanism associated with CME. Although further studies are needed to define the precise role of KIF20A in this pathway, these results provide preliminary evidence for its involvement in membrane invagination or vesicle trafficking during viral entry.

## Discussion

RNA viruses mutate more frequently compared to that of DNA viruses, leading to the continuous emergence of new viral variants (Peck and Lauring, 2018; Visher et al., 2016). This presents a significant challenge for the development of effective antiviral therapies. In this study, we identified a novel role for KIF20A as a proviral host factor for ZIKV life cycle, especially on the viral internalization of entry. Previous research shows the involvement of KIF20A and its regulatory proteins in the life cycle of influenza virus, DENV, HIV, and HCV (Jeon et al., 2022; Madejon et al., 2015; Pérez-Olais et al., 2019; Zhou et al., 2020), highlighting its broader significance across diverse RNA viruses. We demonstrated that the KIF20A inhibitor paprotrain disrupts ZIKV entry on host cells in this study and has previously been shown to inhibit influenza virus life cycle (Jeon et al., 2022). These findings suggest that KIF20A could serve as a broad-spectrum antiviral target for RNA viruses. Furthermore, since DNA viruses share key cellular entry mechanisms with RNA viruses (Miller and Krijnse-Locker, 2008), KIF20A inhibition may also have therapeutic potential against DNA viruses. Inhibition of Plk1—an upstream regulator of KIF20A—is reported to inhibit HBV replication in host cell (Diab et al., 2017). Therefore, KIF20A emerges as a promising antiviral target for RNA and DNA viruses.

Although kinesins are primarily known for their role in viral egress (Döhner and Sodeik, 2005; Jouvenet et al., 2004), previous researches suggest that they may also influence viral entry, including that of HIV (Dharan and Campbell, 2018) and influenza virus infections (Cho et al., 2020). In this study, we examined whether KIF20A inhibition interferes with the early stages of ZIKV infection. KIF20A inhibition significantly inhibited viral attachment and entry, suggesting its potential role in the early stage of ZIKV infection. Given the critical role of the cytoskeletal system in membrane organization, KIF20A inhibition may disrupt the clustering of ZIKV receptors, such as anexelekto and integrins (Meertens et al., 2017; Wang et al., 2020), thereby reducing viral attachment and entry. Additionally, KIF20A inhibition could impair viral uncoating. Previous research has reported that kinesin-1 facilitates efficient uncoating of adenovirus (Strunze et al., 2011) and HIV-1 (Lukic et al., 2014). Therefore, KIF20A may not only facilitate ZIKV attachment and entry but also regulate subsequent stages of the viral life cycle, such as uncoating, by coordinating receptor dynamics and intracellular trafficking. Further studies employing high-resolution imaging or single-virus tracking techniques could provide deeper insights into the precise mechanisms by which KIF20A contributes to these processes.

Our experiments, encompassing siRNA-mediated knockdown, lentivirus-based stable depletion, and pharmacological inhibition, consistently demonstrate that KIF20A is essential for efficient ZIKV infection on host cells. We also observed that ZIKV infection upregulates KIF20A expression in host cells, presumably reflecting the strategy of the virus to hijack host cytoskeletal machinery for efficient infection. In contrast to directing-acting antiviral therapy, host-directed antiviral therapy is considered to minimize antiviral drug resistance (Bekerman and Einav, 2015; Smyk et al., 2022). Therefore, these findings may provide a foundation for developing KIF20A-targeting strategies as a broad-spectrum antiviral approach. Moreover, given that many existing anti-cancer agents target kinesins, repurposing or modifying these compounds could accelerate their development for clinical use.

Our current data were primarily generated from in vitro studies, highlighting the need for future in vivo studies using more biologically relevant models to validate these results. Although paprotrain is a highly selective small molecule inhibitor of KIF20A, its in vivo efficacy remains largely uncharacterized. Further in vivo validation would be essential to determine its therapeutic potential against ZIKV. In addition, as our experiments were conducted primarily in Vero E6 cells, which lack a complete interferon response and do not fully reflect human tissue-specific contexts, the generalizability of our findings to other cell types or in vivo systems may be limited. Future studies incorporating primary human cells or organoid-based models will be crucial to further validate the role of KIF20A in a physiologically relevant setting. Our dynasore co-treatment data (Fig. 7), together with earlier reports linking KIF20A to myosin II guided vesicle traffic (Miserey-Lenkei et al., 2017), suggest at a potential role of KIF20A in the clathrin-mediated endocytic pathway. However, this association remains indirect and warrants further validation using other approaches, such as live-cell imaging and genetic disruption of CME. Nevertheless, our study provides a valuable starting point for understanding the molecular mechanism of KIF20A in ZIKV infection and exploring its potential as a therapeutic target.

Taken together, we have identified a previously unrecognized role of KIF20A in facilitating ZIKV infection and highlighted its potential as a host-directed therapeutic target. These findings also broaden our understanding of viral entry mechanisms, opening new avenues for antiviral research and drug development.

## Supplementary Information

Fig. S1. Chloroquine (CQ) serves as positive control in ZIKV infection assays. (A) Vero E6 cells were treated with paprotrain at concentrations ranging from 100 to 1 µg/ml. After 24 h, cell viability was determined using the trypan blue exclusion assay. (B–E) Vero E6 cells were infected with ZIKV at a multiplicity of infection (MOI) of 0.5 and co-treated with either 50 µM chloroquine (CQ) or 25 µM ribavirin for 18 h. (B, C) Relative mRNA levels of ZIKV NS3 (B) and E (C) normalized to GAPDH.

Fig. S2. KIF20A inhibition reduces the number of ZIKV-infected Huh-7 cells. (A, B) Huh-7 cells were infected with ZIKV at a multiplicity of infection (MOI) of 5 with or without 20 µg/ml paprotrain. At 42 h post-infection (hpi), cells were fixed, permeabilized, and stained with an anti-ZIKV E protein antibody for flow cytometric analysis. (A) Representative histograms illustrating the proportion of ZIKV E–positive (infected) cells. (B) Percentages of infected cells. (C, D) Huh-7 cells were infected with ZIKV at an MOI of 5, with or without 20 µg/ml paprotrain. At 42 hpi, cells were fixed, permeabilized, and stained with DAPI and an anti-ZIKV E protein antibody for immunofluorescence microscopy. (C) Representative images showing infected cells. (D) Quantification from three independent experiments, where infected cells were defined as ZIKV E–positive nuclei co-localized with DAPI, expressed as a percentage of total DAPI-stained nuclei. Values are expressed as the mean ± standard error of the mean (SEM). All experiments were performed at least twice. Asterisks indicate statistically significant differences as determined using an analysis of variance (ANOVA) (^***^P < 0.001, ^****^P < 0.0001).
