# Supplementary material for: Inhibiting kinesin family member 20A disrupts Zika virus entry by blocking internalization
Source: J Microbiol. 2025 Jun 30;63(6):e2503008. doi: 10.71150/jm.2503008 (PMC13577015; doi:10.71150/jm.2503008)
Supplement: Supplementary file 1 [file jm-2503008-Supplementary-Fig-S1.pdf]

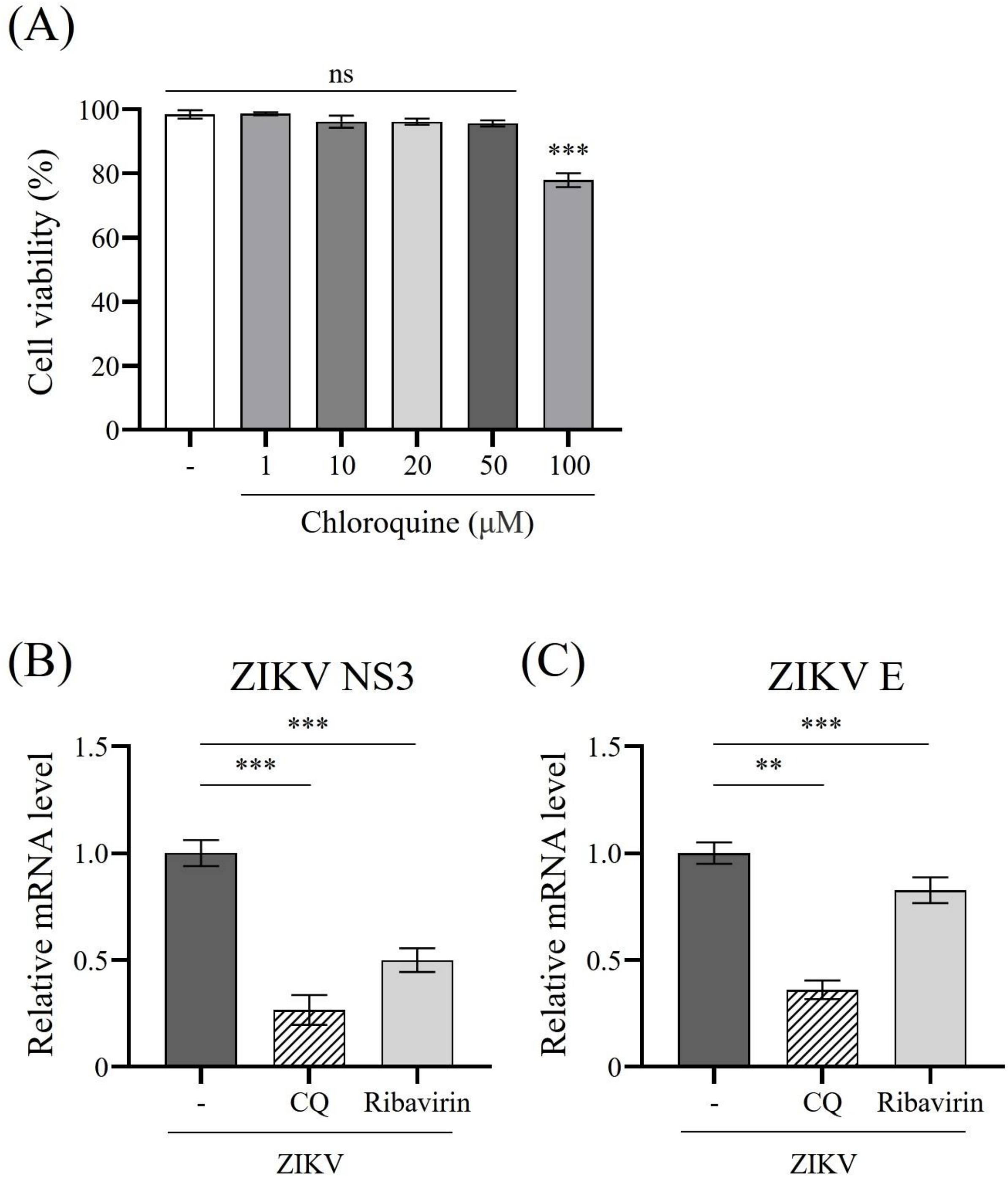

Fig. S1. Chloroquine (CQ) serves as positive control in ZIKV infection assays. (A) Vero E6 cells were treated with paprotrain at concentrations ranging from 100 to 1 μg/ml. After 24 h, cell viability was determined using the trypan blue exclusion assay. (B–E) Vero E6 cells were infected with ZIKV at a multiplicity of infection (MOI) of 0.5 and co-treated with either 50 μM chloroquine (CQ) or 25 μM ribavirin for 18 h. (B, C) Relative mRNA levels of ZIKV NS3 (B) and E (C) normalized to GAPDH.
