# Supplementary material for: Inhibiting kinesin family member 20A disrupts Zika virus entry by blocking internalization
Source: J Microbiol. 2025 Jun 30;63(6):e2503008. doi: 10.71150/jm.2503008 (PMC13577015; doi:10.71150/jm.2503008)
Supplement: Supplementary file 2 [file jm-2503008-Supplementary-Fig-S2.pdf]

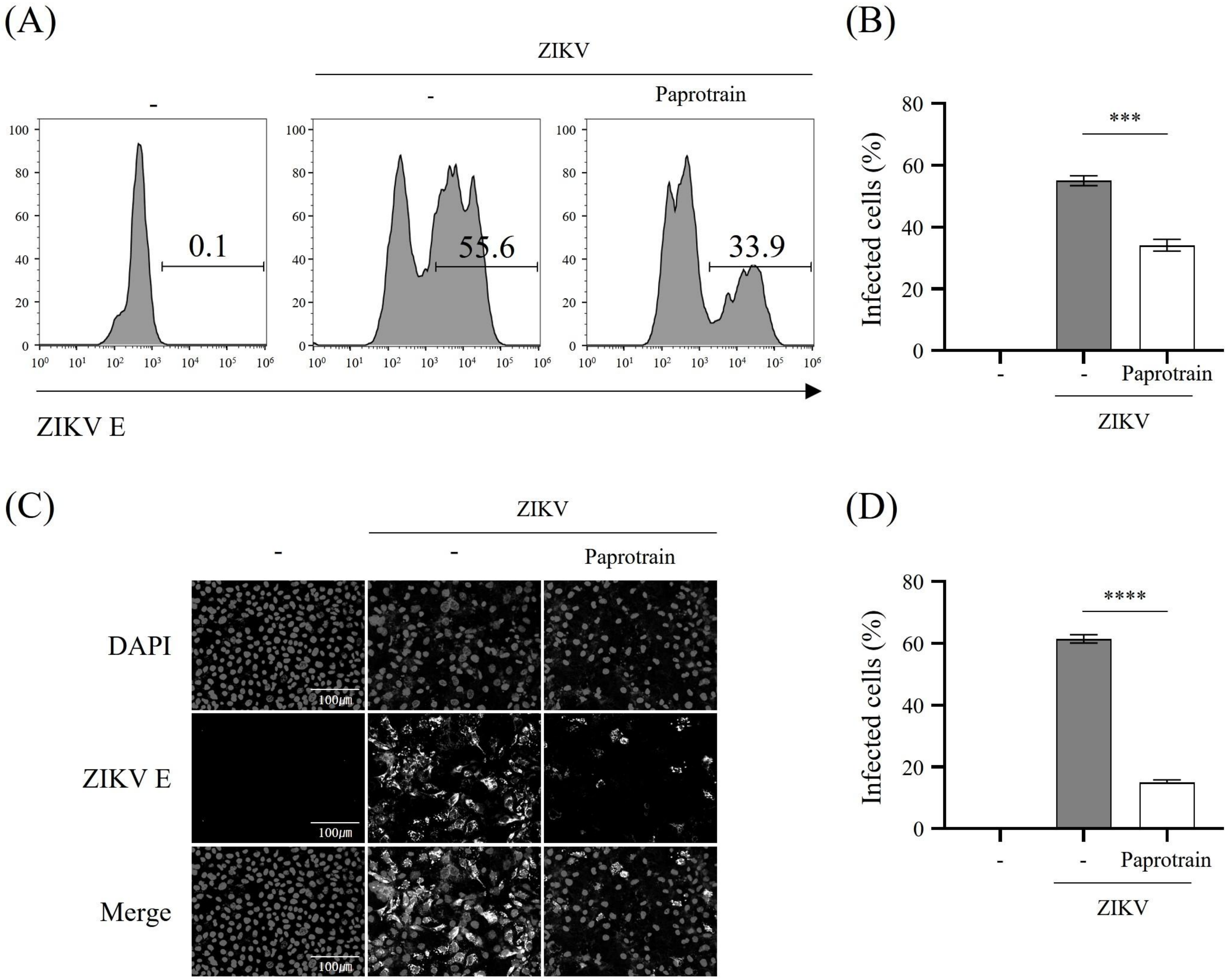

Fig. S2. KIF20A inhibition reduces the number of ZIKV-infected Huh-7 cells. (A, B) Huh-7 cells were infected with ZIKV at a multiplicity of infection (MOI) of 5 with or without 20 μg/ml paprotrain. At 42 h post-infection (hpi), cells were fixed, permeabilized, and stained with an anti-ZIKV E protein antibody for flow cytometric analysis. (A) Representative histograms illustrating the proportion of ZIKV E–positive (infected) cells. (B) Percentages of infected cells. (C, D) Huh-7 cells were infected with ZIKV at an MOI of 5, with or without 20 μg/ml paprotrain. At 42 hpi, cells were fixed, permeabilized, and stained with DAPI and an anti-ZIKV E protein antibody for immunofluorescence microscopy. (C) Representative images showing infected cells. (D) Quantification from three independent experiments, where infected cells were defined as ZIKV E–positive nuclei co-localized with DAPI, expressed as a percentage of total DAPI-stained nuclei. Values are expressed as the mean ± standard error of the mean (SEM). All experiments were performed at least twice. Asterisks indicate statistically significant differences as determined using an analysis of variance (ANOVA) (***$P < 0.001$, ****$P < 0.0001$).
